# Analysis of limb function after various reconstruction methods according to tumor location following resection of pediatric malignant bone tumors

**DOI:** 10.1186/1477-7819-8-39

**Published:** 2010-05-19

**Authors:** Yukihiro Yoshida, Shunzo Osaka, Yasuaki Tokuhashi

**Affiliations:** 1 Department of Orthopedic Surgery, Nihon University School of Medicine, 30-1 Oyaguchikami-cho, Itabashi-ku, Tokyo 173-8610, Japan; 2 Nerima Hikarigaoka Hospital Nihon University, 2-11-1 Hikarigaoka Nerima-ku, Tokyo, Japan

## Abstract

**Background:**

In the reconstruction of the affected limb in pediatric malignant bone tumors, since the loss of joint function affects limb-length discrepancy expected in the future, reconstruction methods that not only maximally preserve the joint function but also maintain good limb function are necessary. We analysis limb function of reconstruction methods by tumor location following resection of pediatric malignant bone tumors.

**Patients and methods:**

We classified the tumors according to their location into 3 types by preoperative MRI, and evaluated reconstruction methods after wide resection, paying attention to whether the joint function could be preserved. The mean age of the patients was 10.6 years, Osteosarcoma was observed in 26 patients, Ewing's sarcoma in 3, and PNET(primitive neuroectodermal tumor) and chondrosarcoma (grade 1) in 1 each.

**Results:**

Type I were those located in the diaphysis, and reconstruction was performed using a vascularized fibular graft(vascularized fibular graft). Type 2 were those located in contact with the epiphyseal line or within 1 cm from this line, and VFG was performed in 1, and distraction osteogenesis in 1. Type III were those extending from the diaphysis to the epiphysis beyond the epiphyseal line, and a Growing Kotz was mainly used in 10 patients. The mean functional assessment score was the highest for Type I (96%: n = 4) according to the type and for VFG (99%) according to the reconstruction method.

**Conclusion:**

The final functional results were the most satisfactory for Types I and II according to tumor location. Biological reconstruction such as VFG and distraction osteogenesis without a prosthesis are so high score in the MSTS rating system. Therefore, considering the function of the affected limb, a limb reconstruction method allowing the maximal preservation of joint function should be selected after careful evaluation of the effects of chemotherapy and the location of the tumor.

## Background

Children who undergo limb-sparing surgery for malignant bone tumors of the lower limbs will face various problems postoperatively as they grow. In particular, limb-length discrepancies and loosening involving a tumor prosthesis can cause serious limb dysfunction. After the resection of malignant tumors in children, a variety of reconstructive procedures have been used on a case-by-case basis, including rotation-plasty [1-5], arthrodesis, bone-lengthening [6-8], extendable prostheses [9-13], extracorporeal irradiated autografts [14-17], vascularized or non-vascularized grafts [18], pasteurization [19], autoclaved bone [20], and amputations [21]. In general reconstructive procedures have been chosen depending on the site of tumor growth, effectiveness of chemotherapy, and predicted limb function. In this study, we classified pediatric malignant bone tumors encountered at our department into 3 types according to the location of the tumor by preoperative MRI, and organized affected limb reconstruction methods after wide resection.

## Methods

We assessed 31 pediatric malignant bone tumor cases treated using lower limb-salvage surgery in our department between 1973 and 2008. The mean age of the 31 patients (16 boys, 15 girls) was 10.6 years (range: 5-15 years), and the mean follow-up was 6 years and 3 months (range: 1-16 years). Enneking's surgical stage was IIB in 30 cases, and IA in one (grade I chondrosarcoma, n = 1). Histological diagnoses were osteosarcoma (n = 26), Ewing's sarcoma (n = 3), primitive neuroectodermal tumor (PNET; n = 1), and grade I chondrosarcoma (n = 1). All but the patients with chondrosarcoma received preoperative chemotherapy. The 3 patients with Ewing's sarcoma, one patient with PNET, and 10 with osteosarcoma were treated jointly with the Pediatric Department. All patients with Ewing's sarcoma and PNET received preoperative radiotherapy for local control (Additional files 1 and 2).

### Operative treatment

Tumors were removed with a new evaluation method for the surgical margin reported by Kawaguchi et al [22]. According to this method, in the case of low-grade sarcoma, obtaining a sufficiently wide margin is essential, but partial margins are acceptable at sites where barriers exist, but a margin greater than 3 cm wide is necessary when preoperative treatment is not conducted or is ineffective in high-grade sarcoma.

### Tumor location

**Figure 1:**
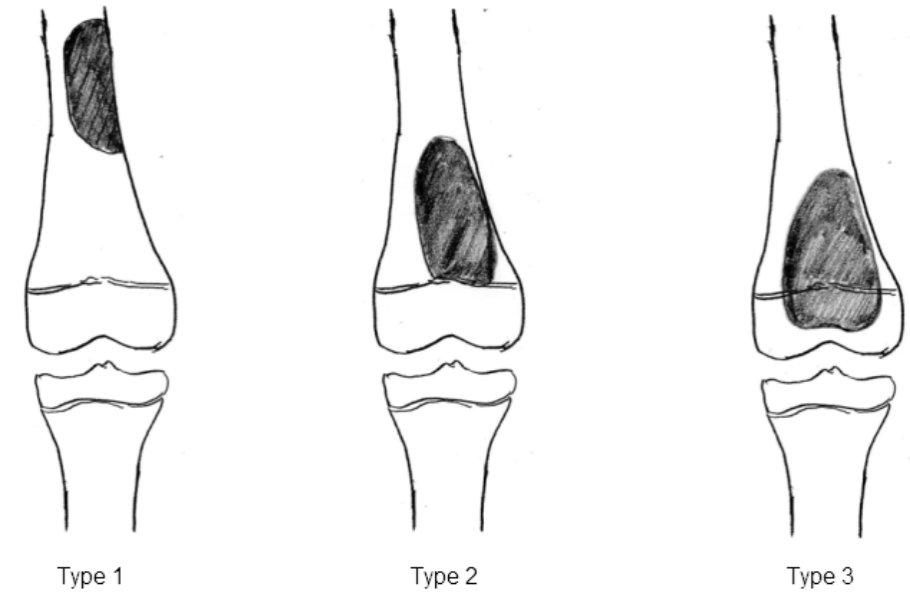
**Classification of tumor location accoding to preoperative MRI**.

The location of the tumor was most frequently the distal femur (15 patients), followed by the proximal tibia (7), proximal femur (6), femoral diaphysis (2), and tibial diaphysis (1). The extension of these tumors was classified by preoperative diagnostic imaging techniques, mainly MRI, into 3 types (Figure 1). The extension of the tumor was evaluated on T1-weighted, T2-weighted, and Gd-enhanced T1-weighted MRI images in coronal, sagittal, and axial planes [23-27] (Figure 1).

#### 1. Type I

Type I tumors were those located in the diaphysis at a distance of ≥ 5 cm from the epiphyseal line. There were 4 patients with this type, and the pathological diagnosis was Ewing's sarcoma in 2 patients, osteosarcoma in 1, and chondrosarcoma in 1. Reconstruction was performed using a vascularized fibular graft.

#### 2. Type II

Type II tumors were those located in contact with the epiphyseal line or within 1 cm from this line. There were 3 patients with this type, of whom 1 showed Type II complicated by Type I. The pathological diagnosis was osteosarcoma in all patients. Reconstruction was performed by VFG in 1 patient and distraction osteogenesis using external fixation in 1. In the other patient with Types I + II (Patient 7), an expandable prosthesis (Lewis type) was used.

#### 3. Type III

Type III tumors were those extending from the diaphysis to the epiphysis beyond the epiphyseal line. This type was the most frequently observed (24 patients). The pathological diagnosis was osteosarcoma in 22 patients, Ewing's sarcoma in 1, and PNET in 1. Reconstruction was performed using a Growing Kotz as an expandable prosthesis in 10 patients, the Howmedica modular reconstruction (HMRS) system as a tumor type prosthesis in 4, Kotz modular femur and tibia reconstruction (KMFTR) system in 1, Kyocera ceramic spacer in 3, and a PHS type I in 2. Rotation-plasty was performed in 4 patients.

All 31 patients were assessed using the revised Musculo-Skeletal Tumour Society (MSTS) rating system [28], complications, limb-length discrepancy, radiological evaluation of prostheses (ISOLS) [29], and outcomes.

## Results

### Functional evaluation

**Figure 2:**
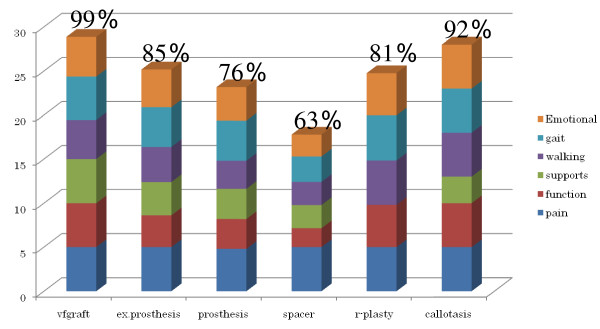
**Functional score by Enneking's functional evaluation**.

The score ranged from 88 to 100% (mean, 96%) for Type I (n = 4), from 76 to 100% (mean, 88.6%) for Type II (n = 3), and from 42 to 100% (mean: 77.4%) for Type III (n = 24). When the score was evaluated according to the reconstruction method, according to the revised Musculo-Skeletal Tumour Society (MSTS) rating system, the overall score for patients undergoing reconstruction with prosthetic joints was only 76%, because the gait score for this group was low due to the knee braces that some patients had to wear. In patients undergoing reconstruction with the Kyocera ceramic spacer, the overall score was also low, at just 63%, since pain and gait scores were low. With patients undergoing reconstructive operations of other types, the overall score was above 89%, and was thus satisfactory. For patients undergoing rotation-plasty, although tests were performed in those wearing lower limb prostheses, scores for gait, walking, and function were all 100%, and the overall functional score was 81% (Figure 2).

### Complications

Postoperative courses were complicated by infection in 2 cases (Cases 16 and 28) skin necrosis in 2 (Case 8 and Case 6), and fracture of the stem of a prosthetic component in one (Case 30). An 8-year-old boy with tibial osteosarcoma (Case 16) underwent reconstruction with a Growing Kotz implant. This patient underwent 1-stage revision at 18 months postoperatively. However, since the infection did not subside, above-knee amputation was performed. A 15-year-old boy treated for osteosarcoma of the left distal femur (Case 28) developed an infection 3 years postoperatively. Despite continuous irrigation and hyperbaric oxygen therapy, the infection persisted. The prosthetic joint was subsequently removed and the joint space was packed with cement beads, but the infection could not be controlled. Rotation-plasty eventually became necessary. Skin necrosis occurred in 2 patients. One was an 8-year-old boy with an osteosarcoma of the proximal tibia (Case 6), and the other was a 5-year-old boy with an osteosarcoma of the distal femur (Case 8). In Case 6, the affected limb was reconstructed by external fixation using the Ilizarov technique after wide resection of the tumor. Partial skin necrosis occurred postoperatively at the insertion site of one of the pins. In Case 8, the skin became partially necrotic at the frontal aspect of the knee. Both patients were treated using plastic surgery. In an 11-year-old boy with osteosarcoma of the distal femur (Case 30), limb reconstruction was performed using the physio-hinge type I system after tumor resection, but the stem of the femoral component was fractured at the base 6 years postoperatively. This patient underwent secondary reconstruction using the physio-hinge type II system. Fortunately, We have no complication about VFG.

### Limb-length discrepancy

During the course, limb-length discrepancy was observed in 10 patients, of whom 7 required treatment. An expandable prosthesis was used in 5 patients, in whom bone lengthening was performed 1-3 times (mean: 2.4 times) when the limb-length discrepancy became 10-20 mm (mean: 13 mm). The total lengthening was 10-43.5 mm (mean: 32.3 mm). A patient using a Growing Kotz type (Case 15) underwent revision arthroplasty due to stem loosening of the tibial component. This patient has undergone bone lengthening 3 times to the present, with a total lengthening of 43.5 mm. Even at present, there is a limb-length discrepancy of 20 mm. A patient (Case 7) with a Lewis type expandable prosthesis for sarcoma in the proximal femur underwent bone-lengthening twice, with a total lengthening of 35 mm. At present, 10 years after the operation, the limb-length discrepancy is 40 mm.

In another patient with the physio-hinge type I system (Case 30), the limb-length discrepancy was corrected when stem fracture of the prosthesis was treated. In a 5-year-old girl with Ewing's sarcoma of the tibia who underwent biological reconstruction with VFG (Case2), a limb-length discrepancy of 36 mm was corrected using external fixation techniques. A 20-mm limb-length discrepancy remained as of 4 years postoperatively (Table 1).

**Table 1:** Details of seven patients with limb length discrepancy.

Case	Type of implant or reconstruction methods	Discrepancy before surgery	Elongation methods and Times of Elongantion	Total lengthening	Discrepancy at present
2	VFG	40 mm	External fixation	36 mm	20 mm
7	Extendable prosthesis (Lewis type)	15 mm	2	35 mm	40 mm
15	Extendable prosthesis (Growing Kotz type)	20 mm	3	43.5 mm	20 mm
18	Extendable prosthesis (Growing Kotz type)	10 mm	3	32 mm	10 mm
19	Extendable prosthesis (Growing Kotz type)	10 mm	3	41 mm	41 mm
20	Extendable prosthesis (Growing Kotz type)	10 mm	1	10 mm	10 mm
30	Tumor prosthesis (Physio-hinge type 1)	65 mm	Bone graft at the fracture site	12 mm	60 mm

### Radiographic evaluation of prosthetic joints

Evaluation using the radiological scale of the International Symposium of Limb Salvage (ISOLS) system was performed in 11 patients who underwent reconstruction using a tumor type or expandable prosthesis and could be observed for 3 years or more. The radiographic result for Bone remodeling was excellent in 4 patients, fair in 3, and poor in 4, that for Interface was excellent in 5, good in 1, fair in 4, and poor in 1, and that for Anchorage was excellent in 10 and good in only 1 (Table 2).

**Table 2:** Radiographic results by International Symposium of Limb Salvage system for radiological assessment of prosthesis.

Case	Bone remodeling	Interface	Anchorage
7	Poor	Good	Good
15	Poor	Fair	Excellent
16	Poor	Fair	Excellent
18	Fair	Excellent	Excellent
19	Fair	Poor	Excellent
20	Fair	Fair	Excellent
21	Excellent	Excellent	Excellent
26	Excellent	Excellent	Excellent
28	Excellent	Excellent	Excellent
29	Excellent	Excellent	Excellent
30	Poor	Fair	Excellent

#### Outcome

Nineteen patients have been continuously disease-free (CDF), whereas 11 have died of disease (DOD), with the cause of death being lung metastasis in all. The patient with Ewing's sarcoma (Case 4) developed brain metastasis, and is alive with disease (AWD) at present.

## Discussion

When the affected limb is reconstructed after the resection of a malignant tumor in a child, such reconstruction is associated with a variety of problems, including an expectation of limb-length discrepancy due to postoperative physical growth, measures to be taken to cope with high levels of physical activity in childhood, and problems related to social adaptation. In some pediatric cases, reconstruction of the lower limb must be designed using an approach completely different from that in adult cases. To solve this problem, we attempted to classify reconstruction of the lower limbs into 3 types based on the sites of tumor location on MRI. Type I tumors were those located in the diaphysis. Type II tumors were those in contact with the epiphyseal line, and Type III tumors were those infiltrating the epiphysis beyond the epiphyseal line [24-26]. The first type involves reconstruction of the shaft of a long bone. VFG is considered to be the most useful technique for the reconstruction of long bone shafts in pediatric cases. In our experience, bone defects up to 15 cm in length can be managed using VFG. If VFG is used to reconstruct a femur, whether the graft is strong enough to bear the individual's body weight is critical. To improve the weight-bearing capacity, Toh et al. reported transplantation of a fibular graft folded in two on a vascular pedicle in 1988 [17,18]. With bone defects exceeding 10 cm in length, we usually use VFG with a fibular graft. None of the VFG patients have experienced complications such as bone fracture [30,31]. Alternatives for reconstructing the diaphysis other than VFG include methods such as pasteurization [19], autoclaved bone [20], and extracorporeal irradiation [14-16]. These methods are superior in conforming to bone defects, there is no immune reaction, and the reconstruction of tendons and ligaments is straightforward, but caution is necessary to avoid fracture or infection of the grafted bone. This approach is apparently applicable to reconstruction of the diaphysis, and has no influence on limb length discrepancies associated with malignant bone tumor resection in infancy. For Type II tumors, which are located in the diaphysis in contact with the epiphyseal line, when adjunctive therapies such as chemotherapy are effective, there is a chance of preserving the joint. If joint preservation is possible, from our experience, reconstruction methods such as distraction osteogenesis with external fixation or the use of VFG can be considered. According to Tsuchiya et al., minimal surgery after caffeine-assisted chemotherapy allowed the articular surfaces to be saved, and successful reconstruction with useful limb function was achieved using callotasis by external fixation [6]. Manfrini et al. reported 6 cases with malignant bone tumors of the tibia in which preservation of the articular surface facilitated successful reconstruction with vascularized fibular autografts and massive bone allografting [32]. Here, the problem is the method of evaluating the degree of tumor invasion. Kumta et al. classified osteosarcomas developing around the epiphyseal line into 5 types by MRI, and reported reconstruction methods using bone allografts according to these types [24].

Tsuchiya et al. used plain radiography, angiography, and Tl scintigraphy to assess the overall effects of preoperative chemotherapy. Manfrini et al. performed preoperative magnetic resonance imaging (MRI) to assess the degree of tumor cell invasion of the epiphysis [23,25]. In our 4 patients treated using minimal surgery, bone tumors were of low malignancy (Cases 1 and 6) and relatively small, preoperative MRI clearly excluded tumor invasion to the epiphysis (Case 5), or a good response to chemotherapy was achieved (Case 3). In 2000, Garcia et al. reported 25 osteosarcoma cases treated using preoperative chemotherapy, and radiological and pathohistological examinations demonstrated the invasion of tumor cells up to the epiphyseal plate in 21 of the 25 patients [33-37]. When minimal surgery is performed, the surgical procedure must be designed carefully. Methods of assessing the effects of preoperative chemotherapy and the extent of tumor invasion are also of critical importance, but have yet to be established. With complications, patients undergoing limb-lengthening by callotasis receive postoperative chemotherapy that can result in non-union and weakening of the bone. To strengthen weak bone in our cases, VFG was added, and pin-tract infection subsequently occurred. Appropriate measures to cope with such complications are important for achieving the reconstruction of a functional limb. With a limb-length discrepancy hindering postoperative limb function, a mean 2-cm difference was noted among cases reported by Manfrini et al., but the limb-length discrepancies causing dysfunction of the affected limb eventually disappeared. In one of our patients, the discrepancy had reached 2 cm by 8 years postoperatively, but did not cause overt gait abnormality. The third type includes tumors invading the epiphyses that require an adequately wide resection with margins of at least 3 cm in the surrounding tissue. Malignant tumors of this type are an indication for tumor type and extendable prostheses. To cope with limb-length discrepancies that may develop in the future, an extendable prosthesis is useful. Extendable prostheses are widely accepted as being indicated for tumors growing close to joints in children around 10 years of age, and for whom resection is expected to cause a limb-length discrepancy ≥ 4 cm. We have used Growing-Kotz implants in 4 patients. One of the 4 patients was a 7-year-old girl with PNET of the left distal femur (Case 15). She underwent limb reconstruction with a Growing-Kotz unit, and has undergone limb-lengthening 4 times to make the limb a total of 43.5 mm longer. As of 5 years postoperatively, however, the limb-length discrepancy had reached 20 mm, with a range of motion of 45° during flexion of the knee joint. Apparent stress shielding was recognized around the stem of the femoral component in this patient. According to Schiller et al. [12] and Dominkus et al.[9], to maintain essentially equal leg lengths, limb-lengthening should be administered when the difference reaches 10-20 mm. Based on this policy, limb-lengthening had to be conducted as frequently as 6-25 times/case. To avoid frequent limb-lengthening, prostheses with an automatic elongation feature were developed for 2 patients. Extendable prostheses of all types are used for the reconstruction of lower limbs after wide resection, with at least 3-cm margins, of malignant bone tumors involving the epiphysis and adjacent terminal segment of the long bone shaft. When such an implant system is used, the short-term postoperative limb function is comparable to that achieved with a tumor prosthesis, but various issues have yet to be resolved, including methods of lengthening, infection, and stress-shielding [38,39]. Extendable prostheses were used in 2 patients with malignancies of the proximal tibia. We also attempted to reconstruct an extensor mechanism of the knee joint with the tensor fascia lata, but were unsuccessful. Finally, in lower limb reconstruction with amputation, rotation-plasty and arthrodesis are useful in some cases. These procedures should be considered when a reconstructive procedure is to be chosen. In particular, rotation-plasty can be used when a tumor-free cut end is desired or when a pathological fracture has occurred [1-5]. We employed knee rotation-plasty in one patient with osteosarcoma of the distal femur associated with a pathological fracture. Rotation-plasty permits the concurrent correction of limb-length discrepancies [3]. A prosthetic limb can be more useful than a reconstructed limb, but the appearance remains problematic. Hillman reported that the cosmetic appearance might be the most important disadvantage of rotation-plasty despite good functional and quality-of-life outcomes [4]. Consequently, if a prosthetic limb is chosen, the patient and family should have all information thoroughly conveyed to them.

**Figure 3:**
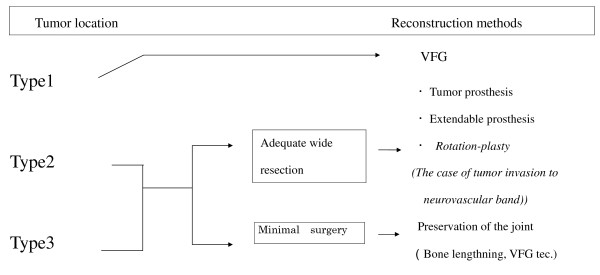
**Guidelines for limb-salvage surgery with resection of malignant bone tumors in children at our department**.

There are various limb reconstruction methods for pediatric malignant bone tumors, and each method has advantages and disadvantages. In this study, we classified such tumors into 3 types according to their location (Figure 3). The final functional evaluation showed the most satisfactory results for Types I and II that allowed joint surface preservation and the maintenance of joint function in the future. However, the joint surface preserving method for these types can be performed only in limited patients who adequately respond to chemotherapy and have a tumor in areas allowing joint surface preservation [40,41]. In the future, it may be necessary to develop adjunctive therapies that have marked effects on Type III, enabling the selection of reconstruction methods similar to those for Type II, and new diagnostic imaging techniques for the evaluation of the effects of such methods.

## Competing interests

The authors declare that they have no competing interests.

## Authors' contributions

YY: carried out operations, supervised statistics, collect data, drafted the manuscript, and acted as corresponding author and did the revisions. YT: was head of the department. SO: carried out operations. All authors read and approved the final manuscript.

## Supplementary Material

Additional file 1 Details of the 31 pediatric patients with limb salvage surgery with resection of malignant bone tumors (Page 1). Click here for file

Additional file 2 Details of the 31 pediatric patients with limb salvage surgery with resection of malignant bone tumors (Page 2). Click here for file
