# Supplementary material for: Analysis of limb function after various reconstruction methods according to tumor location following resection of pediatric malignant bone tumors
Source: World J Surg Oncol. 2010 May 19;8:39. doi: 10.1186/1477-7819-8-39 (PMC2881919; doi:10.1186/1477-7819-8-39)
Supplement: Additional file 1 — Details of the 31 pediatric patients with limb salvage surgery with resection of malignant bone tumors (Page 1). [file 1477-7819-8-39-S1.DOC]

| **Case** | **Age at**  **operation (yrs)** | **Gender** | **Pathological**  **diagnosis** | **Tumor location**  **type** |  | **Resection** | **Reconstruction method** | **Complication** | **Discrepancy** | **Functional score(%)** | **Duration of**  **follow 　up**  **(mos)** | **Prognosis** |
| --- | --- | --- | --- | --- | --- | --- | --- | --- | --- | --- | --- | --- |
| **1** | **12** | **f** | **Chondrosarcoma** | **Ⅰ** |  | **1cm Wide** | **VFG** |  |  | **100** | **84** | **CDF** |
| **2** | **5** | **f** | **Ewing’s sarcoma** | **Ⅰ** |  | **Wide** | **VFG** |  | **20mm** | **96** | **192** | **CDF** |
| **3** | **13** | **m** | **OS** | **Ⅰ** |  | **1cm wide** | **VFG** |  | **10mm** | **100** | **72** | **CDF** |
| **4** | **15** | **m** | **Ewing’s sarcoma** | **Ⅰ** |  | **Wide** | **Prosthesis (HMRS)** |  |  | **88** | **132** | **AWD** |
| **5** | **11** | **f** | **OS** | **Ⅱ** |  | **1cm Wide** | **VFG** |  |  | **100** | **96** | **CDF** |
| **6** | **8** | **m** | **OS** | **Ⅱ** |  | **1cm Wide** | **Callotasis** | **Skin sloughing** | **20mm** | **92** | **150** | **CDF** |
| **7** | **9** | **f** | **OS** | **Ⅰ＋Ⅱ** |  | **Wide** | **Prosthesis (Lewis type)** |  | **40mm** | **76** | **192** | **CDF** |
