# Supplementary material for: Analysis of limb function after various reconstruction methods according to tumor location following resection of pediatric malignant bone tumors
Source: World J Surg Oncol. 2010 May 19;8:39. doi: 10.1186/1477-7819-8-39 (PMC2881919; doi:10.1186/1477-7819-8-39)
Supplement: Additional file 2 — Details of the 31 pediatric patients with limb salvage surgery with resection of malignant bone tumors (Page 2). [file 1477-7819-8-39-S2.DOC]

| **Case** | **Age at operation**  **(yrs)** | **Gender** | **Pathological**  **diagnosis** | **Tumor location**  **type** | **Resection** | **Reconstruction methods** | **Complication** | **Discrepancy** | **Functional**  **score(%)** | **Duration**  **of follow up**  **(mos)** | **Prognosis** |
| --- | --- | --- | --- | --- | --- | --- | --- | --- | --- | --- | --- |
| **8** | **5** | **m** | **OS** | **Ⅲ** | **Wide** | **Spacer** | **Skin sloughing** |  | **42** | **12** | **DOD** |
| **9** | **8** | **m** | **OS** | **Ⅲ** | **Wide** | **Spacer** |  |  | **76** | **19** | **DOD** |
| **10** | **6** | **m** | **OS** | **Ⅲ** | **Wide** | **Spacer** |  |  | **72** | **12** | **DOD** |
| **11** | **13** | **m** | **OS** | **Ⅲ** | **Wide** | **Hip rotation plasty** |  |  | **68** | **12** | **DOD** |
| **12** | **9** | **f** | **OS** | **Ⅲ** | **Wide** | **Knee rotation plasty** |  |  | **100** | **240** | **CDF** |
| **13** | **13** | **f** | **OS** | **Ⅲ** | **Wide** | **Knee rotation plasty** |  |  | **78** | **15** | **DOD** |
| **14** | **12** | **m** | **OS** | **Ⅲ** | **Wide** | **Knee rotation plasty** |  |  | **80** | **96** | **CDF** |
| **15** | **7** | **f** | **PNET** | **Ⅲ** | **Wide** | **Prosthesis (Growing Kotz)** |  | **20mm** | **80** | **132** | **CDF** |
| **16** | **8** | **m** | **OS** | **Ⅲ** | **Wide** | **Prosthesis (Growing Kotz)** | **Infection** |  | **76** | **84** | **CDF** |
| **17** | **12** | **m** | **Ewing’s sarcoma** | **Ⅲ** | **Wide** | **Prosthesis (Growing Kotz)** |  |  | **88** | **24** | **DOD** |
| **18** | **13** | **m** | **OS** | **Ⅲ** | **Wide** | **Prosthesis (Growing Kotz)** |  | **10mm** | **96** | **78** | **CDF** |
| **19** | **8** | **m** | **OS** | **Ⅲ** | **Wide** | **Prosthesis (Growing Kotz)** |  | **41mm** | **96** | **76** | **CDF** |
| **20** | **10** | **m** | **OS** | **Ⅲ** | **Wide** | **Prosthesis (Growing Kotz)** |  | **10mm** | **91** | **93** | **CDF** |
| **21** | **11** | **m** | **OS** | **Ⅲ** | **Wide** | **Prosthesis (Growing Kotz)** |  |  | **96** | **108** | **CDF** |
| **22** | **16** | **f** | **OS** | **Ⅲ** | **Wide** | **Prosthesis (Growing Kotz)** |  |  | **95** | **72** | **CDF** |
| **23** | **12** | **m** | **OS** | **Ⅲ** | **Wide** | **Prosthesis (Growing Kotz)** |  |  | **85** | **70** | **CDF** |
| **24** | **16** | **m** | **OS** | **Ⅲ** | **Wide** | **Prosthesis (Growing Kotz)** |  |  | **55** | **12** | **DOD** |
| **25** | **12** | **f** | **OS** | **Ⅲ** | **Wide** | **Prosthesis (HMRS)** |  |  | **52** | **13** | **DOD** |
| **26** | **13** | **m** | **OS** | **Ⅲ** | **Wide** | **Prosthesis (HMRS)** |  |  | **96** | **60** | **DOD** |
| **27** | **12** | **f** | **OS** | **Ⅲ** | **Wide** | **Prosthesis (KMFTR)** |  |  | **56** | **14** | **DOD** |
| **28** | **15** | **m** | **OS** | **Ⅲ** | **Wide** | **Prosthesis (HMRS)** | **Infection** |  | **52** | **156** | **CDF** |
| **29** | **14** | **f** | **OS** | **Ⅲ** | **Wide** | **Prosthesis (HMRS)** |  |  | **96** | **60** | **CDF** |
| **30** | **11** | **m** | **OS** | **Ⅲ** | **Wide** | **Prosthesis (PHS-1)** | **Stem breakage** | **60mm** | **84** | **180** | **CDF** |
| **31** | **12** | **f** | **OS** | **Ⅲ** | **Wide** | **Prosthesis (PHS-1)** |  |  | **84** | **14** | **DOD** |

Abbreviations: OS: osteosarcoma, PNET: primitive neuroectodermal tumor, df: distal femur, fs: femoral shaft, ts: tibial shaft, pf: proximal femur, Wide: wide resection, HMRS: Howmedica modular resection system; KMFTR: Kotz modular femur and tibia reconstruction system, PHS-1: Physio-hinge type 1, Callotasis: Bone-lengthening by callus distraction, AWD: Alive with disease, CDF: Continuously disease-free, DOD: Died of disease, NED: No evidence of disease
